# Mechanical stretch-induced vascular hypertrophy occurs through modulation of leptin synthesis-mediated ROS formation and GATA-4 nuclear translocation

**DOI:** 10.3389/fphar.2015.00240

**Published:** 2015-10-23

**Authors:** Crystal M. Ghantous, Firas H. Kobeissy, Nadia Soudani, Farah A. Rahman, Mustafa Al-Hariri, Hana A. Itani, Ramzi Sabra, Asad Zeidan

**Affiliations:** ^1^Cardiovascular Physiology Lab, Department of Anatomy, Cell Biology and Physiology, American University of Beirut, Beirut, Lebanon; ^2^Department of Biochemistry and Molecular Genetics, American University of Beirut, Beirut, Lebanon; ^3^Division of Clinical Pharmacology, Department of Medicine, Vanderbilt University School of Medicine, Nashville, TN, USA; ^4^Department of Pharmacology and Toxicology, American University of Beirut, Beirut, Lebanon

**Keywords:** mechanical stretch, leptin, vascular smooth muscle, remodeling, ROS

## Abstract

**Background**: Obesity and hypertension are associated with increased leptin production contributing to cardiovascular remodeling. Mechanisms involving mechanical stretch-induced leptin production and the cross talk between signaling pathways leading to vascular remodeling have not been fully elucidated.

**Methods and Results**: Rat portal vein (RPV) organ culture was used to investigate the effect of mechanical stretch on leptin protein expression in vascular smooth muscle cells (VSMCs). Moreover, the involvement of reactive oxygen species (ROS), the RhoA/ROCK pathway, actin cytoskeleton dynamics and the transcriptional factor GATA-4 activation in mechanical stretch-induced vascular remodeling were investigated. Stretching the RPV for 1 or 24 h significantly increased leptin protein level and ROS formation in VSMCs, which was prevented by 1 h pretreatment with the ROCK inhibitor Y-27632 and the actin cytoskeleton depolymerization agent cytochalasin D. Moreover, Western blotting and immunohistochemistry revealed that mechanical stretch or treatment with 3.1 nmol/L leptin for 24 h significantly increased actin polymerization, as reflected by an increase in the F-actin to G-actin ratio. Increases in blood vessels’ wet weight and [^3^H]-leucine incorporation following a 24 h treatment with conditioned media from cultured stretched RPVs indicated RPV hypertrophy. This effect was prevented by 1 h pretreatment with anti-leptin antibody, indicating leptin’s crucial role in promoting VSMC hypertrophy. As an index of GATA-4 activation, GATA-4 nuclear translocation was assessed by immunohistochemistry method. Pretreating VSMC with leptin for 1 h significantly activated GATA-4 nuclear translocation, which was potently attenuated by the NADPH oxidase inhibitor apocynin, Y-27632, and cytochalasin D.

**Conclusion:** Our results demonstrate that ROS formation, RhoA/ROCK pathway, and GATA-4 activation play a pivotal role in mechanical stretch-induced leptin synthesis leading to VSMC remodeling.

## Introduction

The cardiovascular system is constantly exposed to mechanical stress due to blood pressure. An increase in blood pressure (hypertension) leads to vascular and cardiac remodeling (Zeidan et al., 2003b, 2007; Zhang et al., 2005; Kai et al., 2009). Hypertension, regardless of its underlying cause, is a major risk factor for other cardiovascular diseases, such as vascular hypertrophy, left ventricular hypertrophy, and heart failure (Zeidan et al., 2003b; Zhang et al., 2005). Different signaling pathways are involved in hypertension-induced vascular remodeling (Zeidan et al., 2003b; Zhang et al., 2005) such as the RhoA/ROCK pathway which results in an increased in actin polymerization and depletion in G-actin (Zeidan et al., 2007). Under normal physiological conditions, the ratio of F-actin to G-actin is low, thus attenuating the activity of the transcription factor SRF and down-regulating hypertrophic gene expression (Nelson et al., 2005; Zeidan et al., 2006). However, activation of the RhoA/ROCK pathway increases F-actin to G-actin ratio and as a result, promotes vascular remodeling and hypertrophy. PI3K/AKT pathway is another pathway that is involved in the changes in actin cytoskeleton dynamics via LIMK/cofilin phosphorylation (Zeidan et al., 2007). Thus there is a cross-talk between the PI3K/AKT and RhoA/ROCK pathways in promoting vascular hypertrophy (Li et al., 2009; Fan et al., 2014). The activation of the MAP kinases ERK1/2 and p38 is also another essential pathway involved in mechanical stretch-induced VSMC hypertrophy (Zeidan et al., 2000).

Hypertension is associated with high levels of the obesity-associated protein leptin (hyperleptinemia; Agata et al., 1997; Uckaya et al., 1999). In addition, we have previously shown that mechanical stretch, which mimics hypertension, enhances the release of leptin from vascular smooth muscle cells (VSMCs; Zeidan et al., 2005). Leptin is a 16 kDa protein that is best known for its function as a satiety factor. It circulates in the blood of lean individuals at levels between 5 and 15 ng/mL (Sinha et al., 1996). These levels are higher in obese individuals and may reach up to 50 ng/mL, due to a higher adipose tissue mass (Sinha et al., 1996). In addition to adipocytes, leptin is produced by other kinds of cells including cardiomyocytes and VSMCs (Zeidan et al., 2005; Matsui et al., 2007). Different studies have shown that the functional leptin receptor, leptin receptor b (OBRb), is found in a variety of cells such as VSMCs (Zeidan et al., 2005; Zeidan and Karmazyn, 2006), cardiomyocytes (Rajapurohitam et al., 2003), myometrial cells (Markowska et al., 2005), endothelial cells (Mutze et al., 2006), and T-lymphocytic cells (Martin-Romero et al., 2000; Lord et al., 2002). Therefore, this hormone has a wide range of pleiotropic effects and affects the cardiovascular, nervous, reproductive, and immune systems (Lord et al., 1998; Margetic et al., 2002; Rahmouni and Haynes, 2004). Leptin promotes hypertension, vascular remodeling, sympathetic nervous system stimulation, ROS generation, angiogenesis, and atherosclerosis (reviewed, by Ghantous et al., 2015). Hence, leptin potentially contributes to many cardiovascular risks associated with obesity.

Blood vessels exposed to hypertension generate excessive levels of reactive oxygen species (ROS; Paravicini and Touyz, 2006, 2008; Montezano and Touyz, 2012). The main source of cardiovascular ROS is a family of NADPH oxidases (Nox), such as Nox1, Nox2, Nox4, and Nox5 (Montezano and Touyz, 2012). Under stressful environmental conditions like hypertension, ROS levels increase in a phenomenon known as oxidative stress (Montezano and Touyz, 2012). ROS-mediated endothelial cell and VSMC damage promotes remodeling of the vasculature and atherosclerosis (Touyz and Schiffrin, 2004; Mei et al., 2015).

The transcription factor GATA-4 has been studied in the cardiac system to promote hypertrophy by translocating to the nucleus and activating hypertrophic gene expression, such as β-MHC, c-fos, c-jun, c-myc, BNP, and ANF (Chien et al., 1991; Liang and Gardner, 1999; Saadane et al., 1999; Babu et al., 2000; Hsieh et al., 2015). For example, GATA-4 has been shown to modulate the transcriptional activation of angiotensin II type_1*A*_ receptor and β-MHC in pressure overload-induced cardiac hypertrophy (Herzig et al., 1997; Oka et al., 2006). GATA-4 also interacts with NFAT3, another transcription factor that is involved in promoting cardiac hypertrophy (Molkentin et al., 1998). Thus, GATA-4 has been well characterized in the cardiac system in promoting hypertrophy; however, the present knowledge of the role it plays in the vascular system has been limited to regulating cardiac angiogenesis (Heineke et al., 2007) and embryonic angiogenesis in the vascular plexus (Torregroza et al., 2012). Since GATA-4 is well known to promote cardiac hypertrophy, it is interesting to study whether leptin-induced vascular remodeling could be mediated by GATA-4, thus discovering a new role for GATA-4 in the vascular system.

The mechanisms by which hypertension/leptin or ROS induce vascular hypertrophy have not been fully elucidated yet. Accordingly, the present study was designed to determine the interaction between hypertension, leptin, and ROS and to identify their role in promoting vascular hypertrophy. In addition, the involvement of the RhoA/ROCK pathway and actin cytoskeleton remodeling in hypertension/leptin-induced ROS formation and vascular hypertrophy were investigated in this study. The transcriptional factor GATA-4 nuclear translocation was also examined in order to take a detailed look at the mechanism promoting VSMC hypertrophy.

To achieve these aims, rat portal vein (RPV) organ culture was used. This blood vessel is a large vessel that is both pre-and post-capillary and has spontaneous myogenic tone (Sutter, 1990; Zeidan et al., 2000). Being a low-pressure vessel, it is very sensitive to pressure increase (Malmqvist and Arner, 1988, 1990). Moreover, the RPV has been extensively used as an analog of small pre-capillary resistance vessels (Ljung, 1990; Sutter, 1990). Morphologically, the portal vein has a thin inner circular layer of VSMCs and a thick, very pronounced longitudinal layer of VSMCs (Sutter, 1990). The longitudinal orientation of the VSMCs provides an *in vitro* model to mimic hypertension by mechanically stretching it using weights that stretch the vein equivalently to the *in vivo* force of stretch exerted on blood vessels in hypertension (see Rat Portal Vein Organ Culture).

## Materials and Methods

### Rat Portal Vein Organ Culture

Male Sprague-Dawley rats (200–250 g) were killed by CO_2_, as approved by the Animal Ethics Committee, American University of Beirut. The investigation conforms to the Guide for the Care and Use of Laboratory Animals published by the US National Institutes of Health (NIH Publication No. 85–23, revised 2011). RPVs were dissected in a sterile environment and cleaned in N-Hepes buffer solution (400 mM NaCl, 200 mM KCl, 100 mM MgCl2, 100 mM Hepes, 11.5 mM Glucose, 5% penicillin-streptomycin). RPVs were cut longitudinally resulting in two equal portal vein strips and weighed when needed. Silver weights of 0.6 g (stretch the blood RPV slightly above optimal length or 10% stretch) were tied to the end of the strip on which stretch studies were to be performed. The other strip was left unstretched and used as a control. In cases where an entire vein was needed, weights of 1.2 g were used. The strips were then transferred to culture media of DMEM/F-12 HAM with 5% penicillin/streptomycin). RPVs were incubated at 37°C and 5% CO_2_ in air. When leptin (Rat Leptin, Biovision, San Francisco, USA) was used as treatment, a concentration of 3.1 nmol/L was added.

Inhibitors such as the selective ROCK inhibitor Y-27632 (10 μmol/L, Sigma Aldrich, MO, USA), the actin depolymerization agent cytochalasin D (1 μmol/L; Calbiochem, CA, USA), the NADPH oxidase inhibitor apocynin (1 μmol/L, 4-Hydroxy-3-methoxyacetophenone, Sigma Aldrich, MO, USA), and the anti-leptin antibody Ob (Y-20) (1 μmol/L; Santa Cruz Biotechnology, CA, USA) were added to the media 60 min before mechanically stretching the RPV or adding leptin. Following incubation, the RPV strips were taken out of the incubator and either weighed (Zeidan et al., 2000) or immediately frozen in liquid nitrogen and stored at –80°C for protein analysis.

When conditioned media (CM) was used, RPVs were cultured in DMEM F-12 HAM either unstretched or stretched for 24 h. Media was then centrifuged at 800 × *g* for 10 min, followed by centrifugation at 5000 RPM for 15 min with a Centricon Plus-20 filter (Millipore Corporation, MA, USA) to concentrate the media 100-fold and yield concentrated CM.

### Immunoblotting

Rat portal veins were homogenized in 100 μL of lysis buffer (50 mM Tris, pH 8.0, 150 mM NaCl, 1% Nonidet-P40, 0.5% sodium deoxycholate) using liquid nitrogen. Proteins were extracted after centrifugation for 10 min at 4°C and quantified using Bradford assay. Protein expression and phosphorylation were determined by Western blot as previously described (Zeidan et al., 2006). Primary antibodies for leptin [anti-leptin antibody Ob (Y-20), Santa Cruz Biotechnology, CA, USA], p-GATA-4 (Ser 262, Santa Cruz Biotechnology, CA, USA), and GAPDH (Santa Cruz Biotechnology, CA, USA) were added to the membranes at 1:1000 ratio with 3% BSA for 1 h.

### Measurement of G-actin/F-actin Ratio

F-actin and G-actin were extracted and blotted as described previously (Zeidan et al., 2007). Briefly, RPVs were snap-frozen and homogenized in F-actin stabilization buffer [50 mmol/L PIPES, 5 mmol/L MgCl2, 50 mmol/L NaCl, 5 mmol/L EGTA, 5% (v/v) lyceral, 0.1% (v/v) Triton X-100, 0.1% (v/v) Nonidet P-40, 0.1% (v/v) Tween 20, 0.1% (v/v) 2-mercaptoethanol and 0.001% (v/v) antifoam and a protease inhibitor cocktail] followed by high speed centrifugation for 1 h at 100,000 × *g* to separate the G-actin (supernatants) from F-actin (pellets). In order to depolymerize the F-actin, the pellets were resuspended in cold cytochalasin D (1 μmol/L; Calbiochem, CA, USA). Equal amounts of both the supernatant and the resuspended pellet were subjected to Western blot analysis using rabbit polyclonal actin antibody (Cell Signaling Technology, MA, USA).

### Measurement of Protein Synthesis

Rat portal veins were kept in organ culture with the different kinds of CM for 2 days. RPVs were then cultured for another 24 h with [^3^H]-leucine in order to measure protein synthesis as described previously (Zeidan et al., 2003b).

### RNA Isolation and Real-time PCR

RNA was isolated and Real-Time PCR was done as previously described (Zeidan et al., 2005). The primers were: Nox1 forward 5′-TTTCCTAAACTACCGACTC-3′ and Nox1 reverse 5′-GTGCGACAACGGACTATC-3′, Nox2 forward 5′-CCCTTTGGTACAGCCAGTGAAGAT-3′ and Nox2 reverse 5′-CAATCCCAGCTCCCACTAACATCA-3′, Nox4 forward 5′-GGATCACAGAAGGTCCCTAGCAG-3′ and Nox4 reverse 5′-GCAGCTACATGCACACCTGAGAA-3′, and 18S rRNA forward 5′-GTAACCCGTTGAACCCCATT-3′ 18S rRNA reverse 5′-CCATCCAATCGGTAGTAGCG-3′ which was used as the housekeeping gene to normalize expression.

### ROS Analysis

Reactive oxygen species production in response to mechanical stretch or leptin was detected by dihydroethidium (DHE) staining (10 μM, Sigma-Aldrich). RPV slices (5 μm-thick frozen sections) were incubated with DHE at 37°C, 5% CO_2_ for 30 min in a humidified chamber protected from light. Positive DHE intensity signals were quantified using a laser confocal microscope (LSM710, ZEN confocal software Carl Zeiss).

### Immunohistochemistry

To visualize leptin expression, 5 μm-thick frozen RPV sections were fixed using 4% formaldehyde and permeabilized using 0.2% Triton X-100. Nonspecific binding was blocked by 1% BSA, 0.1% Triton x-100 in PBS for 10 min followed by incubation with anti-leptin antibody [Ob (Y-20) at 1:100 ratio in 1% BSA and 0.05% Tween in PBS] for 1 h. RPV sections were then washed and incubated for another hour at room temperature with Alexa 594-conjugated goat anti-mouse secondary antibody (1:250 in 1% BSA and 0.05% Tween in PBS; Molecular Probes). Images were acquired with a laser confocal microscope (LSM710, ZEN confocal software Carl Zeiss).

For F-actin and G-actin ratio study, RPV frozen sections were fixed in 4% formaldehyde, 0.2% Triton x-100 in the cytoskeleton stabilizing PEM buffer (100 mM PIPES, 5 mM EGTA, 2 mM MgCl2, pH 6.9) for 20 min at room temperature. Thereafter, the sections were permeabilized, blocked, and stained with the F-actin stain Phalloidin (100 nM; Acti-stain 555 phalloidin, Cytoskeleton, Denver, CO, USA) and the G-actin stain Deoxyribonuclease I (300 nM; Alexa Fluor 488 conjugate, Invitrogen, NY, USA). All sections were examined and positive intensity signals were quantified using a laser confocal microscope (LSM710, ZEN confocal software Carl Zeiss).

### Detection of GATA-4 Nuclear Translocation

Rat Aortic Smooth Muscle Cells (RASMC) were cultured at a concentration of 40 × 10^3^ per ml in DMEM media supplemented with 10% fetal bovine serum for 72 h and then starved for another 24 h. Leptin (3.1 nmol/L) was added with or without Y-27632 (10 μmol/L), cytochalasin D (1 μmol/L), or apocynin (1 μmol/L). Cells were then rinsed twice with PBS without Ca^2+^/Mg^2+^, fixed with 4% paraformaldehyde for 10 min, permeabilized with 0.2% Triton X-100 for 20 min, and blocked with 1% BSA, 0.1% Triton x-100 in PBS for 1 h. Anti-GATA-4 antibody (GATA-4, Santa Cruz Biotechnology, CA, USA) at 1:100 ratio in 1% BSA, 0.05% Tween in PBS was incubated with the RASMC overnight at 4°C, followed by incubation with CruzFluor 488-conjugated goat anti-mouse secondary antibody (1:250 in 1% BSA and 0.05% Tween in PBS) in the dark for 1 h. Phalloidin (100 nM; Acti-stain 555 phalloidin, Cytoskeleton, Denver, CO, USA) was then added for 20 min to stain actin. Images were acquired using a laser confocal microscope (LSM710, ZEN confocal software Carl Zeiss).

### Determination of Leptin Release

To determine whether mechanical stretch stimulates leptin release from VSMCs, culture medium was assayed for leptin protein by using a TiterZyme enzyme immunometric assay kit (Assay Designs, Inc., Ann Arbor, MI, USA).

### Statistical Analysis

Values for the experimental groups were normalized to the unstretched RPVs. Data values are presented as mean ± standard error of the mean (SEM). Statistical data was analyzed using *t*-test or one-way analysis of variance (ANOVA), and significance was established by Holm-Sidak or Tukey methods. Statistical significance was considered for *p*<0.05 between groups.

## Results

### Mechanical Stretch/hypertension Increases the Intracellular Leptin Protein Levels in VSMCs

We have shown previously the ability of mechanical stretch to induce leptin secretion into culture media after 1–3 days of stretching (Zeidan et al., 2005). In this study, we investigated the effect of mechanical stretch on the endogenous leptin expression level in VSMCs. RPVs were cultured for 1 h or 24 h with or without stretch.

**FIGURE 1:**
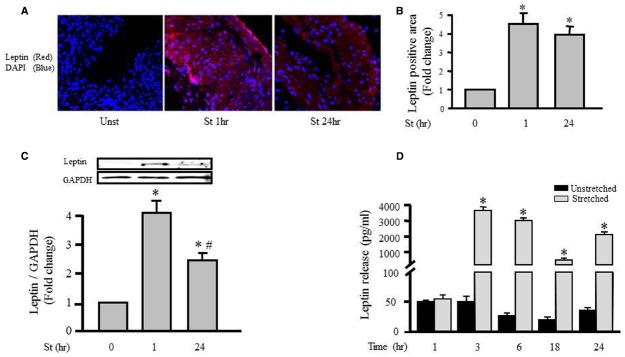
**Mechanical stretch-induced intracellular leptin protein expression and secretion.** RPVs were subjected to stretch for 1 or 24 h **(A)**. Representative confocal microscopic images for leptin detection in RPV wall after stretching for 1 or 24 h. DAPI stained the nuclei blue, while leptin primary antibody was visualized by Alexa 594-conjugated secondary antibody (red). **(B)**. Fluorescence intensity measurements of leptin protein detected by immunohistochemistry. **(C)**. Expression of leptin protein was evaluated using Western blotting densitometric scans and normalized to the unstretched RPVs. (*n*=5–9). Stretch for 1 h significantly increased intracellular leptin expression, more than stretch for 24 h. **(D)**. Leptin release into the extracellular media was measured using ELISA for mechanically stretched RPVs for 1, 3, 6, 18, or 24 h. Leptin release significantly increased after 3–24 h of stretch. *p < 0.05 versus unstretched (Unst). #*p* < 0.05 versus stretched 1 h (St 1 h).

The effect of mechanical stretch on leptin expression in VSMCs was first analyzed using a laser confocal microscope (LSM710, ZEN confocal software, Carl Zeiss). Figures 1A,B show that intracellular leptin levels were significantly increased after 1 and 24 h of mechanical stretch. These findings indicate that mechanical stretch has the ability to induce leptin protein synthesis at early (1 h) and late stages (24 h). In agreement with the previous study of immunohistochemistry, Western blot analysis of protein lysates prepared from the RPVs revealed that intracellular leptin expression was significantly increased after 1 h of mechanical stretch by fourfold and after 24 h of stretch by 2.5 fold (Figure 1C).

To explore the role of mechanical stretch-induced leptin expression on leptin release from VSMCs into the culture medium, we examined the effect of mechanical stretch for 1, 3, 6, 18, and 24 h on leptin release using an immunometric assay. Figure 1D shows that stretching RPVs for 3, 6, 18, or 24 h significantly induced leptin release into the culture medium. These results indicate the direct effect of mechanical stretch on both leptin synthesis and release.

### Role of Endogenous Leptin in Mediating Stretch-induced Hypertrophy

**FIGURE 2:**
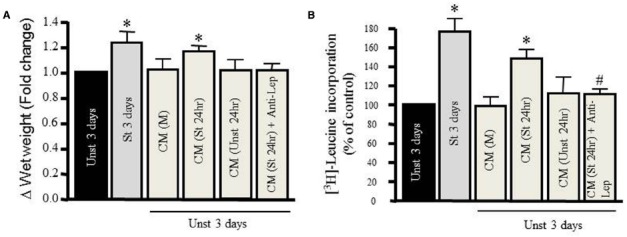
**Mechanical stretch-induced VSMC hypertrophy is mediated by leptin protein release.** Effect of different kinds of conditioned media (CM) prepared from either media alone (M), from unstretched (Unst) RPVs’ media (Unst 24 h), from stretched RPVs’ media (St 24 h), or CM from stretched RPVs with anti-leptin antibody [CM(St 24 h) + Anti-Lep] on tissue wet weight **(A)** and [^3^H]-leucine incorporation **(B)** in RPVs cultured for 3 days. *p < 0.05 versus unstretched (Unst). (*n*=6–9). #*p* < 0.05 versus stretched 24 h (St 24 h).

We tested the hypertrophic effect of CM taken from RPVs unstretched or stretched for 24 h. The CM removed from unstretched RPVs [CM (Unst 24 h)] had no effect on wet weight or protein synthesis of RPVs cultured for 3 days with the CM (Figures 2A,B). However, CM taken from stretched RPVs [CM (St 24 h)] significantly increased RPV wet weight (Figure 2A) as well as protein synthesis (Figure 2B). We also determined the relationship between wet and dry RPV weights in control and hypertrophied strips (data not shown); there were no significant differences in the dry weight/wet weight ratios in different groups, indicating that increased tissue weights were not due to increased water retention.

To ascertain whether CM-induced hypertrophy was mediated by leptin, RPVs were cultured for 3 days with anti-leptin antibody (1 μmol/L) and CM taken from stretched RPVs for 24 h [CM (St 24 h) + Anti-Lep]. As shown in Figures 2A,B, treatment with anti-leptin antibody significantly attenuated CM-induced increase in tissue weight and protein synthesis, suggesting that the hypertrophic effect of CM is mediated, at least in part, by leptin in CM.

### Mechanical Stretch- and Leptin-induced Actin Cytoskeleton Remodeling

**FIGURE 3:**
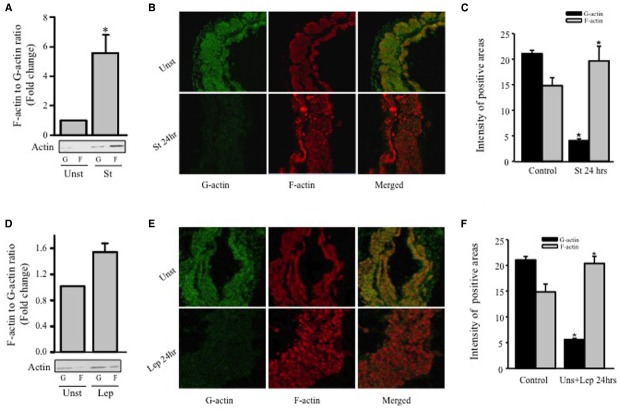
**Mechanical stretch- and leptin- induced actin cytoskeleton dynamics.** RPVs were cultured for 24 h under mechanical stretch **(A–C)** or treated with 3.1 nmol/L leptin **(D–F)**. F- and G-actin were separated by ultracentrifuge followed by Western blot of pellets (F-actin; F) and supernatants (G-actin; G). (*n*=5). *p < 0.05 versus unstretched (Unst). Representative confocal microscopic images for RPV wall stained with the G-actin stain Deoxyribonuclease I **(B,E)**; (left panels; green) and the F-actin stain Acti-stain 555 phalloidin **(B,E)**; (middle panels; red). The overlay of both F- and G- actin (merged) is shown in the right panels **(B,E)**. (*n*=3). Quantification of Phalloidin and Deoxyribonuclease I positive areas indicate that mechanical stretch **(C)** or leptin **(F)** for 24 h resulted in a decrease in G-actin levels and increase in F-actin levels.

Since hypertrophy is characterized by remodeling of the actin cytoskeleton, Western blot analysis was performed for G-actin and F-actin in order to study the effect of mechanical stretch on hypertrophy. RPVs were mechanically stretched for 24 h, and the ratio of F-actin to G-actin was calculated and normalized to the unstretched (control) RPVs (Figure 3A). The F/G-actin ratio for the stretched RPVs was significantly greater (approximately 5.6 fold) than that for the control RPVs (Figure 3A), indicating that the levels of F-actin had increased significantly relative to those of G-actin, a main indicator of hypertrophy (Figure 3).

Rat portal veins were treated with leptin at a concentration of 3.1 nmol/L (50 ng/mL) due to its equivalence to leptin concentrations in obesity (Maffei et al., 1995; Rajapurohitam et al., 2003; Zeidan et al., 2005) for 24 h and the ratio of F/G-actin was calculated and normalized to unstretched (untreated) RPVs (Figure 3D). Similarly to the effect of mechanical stretch, treatment with leptin resulted in a significant increase in F/G-actin ratio (approximately 1.5 fold), indicating that leptin alone indeed promotes vascular remodeling in the form of hypertrophy.

The effect of mechanical stretch and leptin on actin cytoskeleton remodeling of RPVs was also analyzed by confocal microscopy using Phalloidin and Deoxyribonuclease I to stain F-actin and G-actin, respectively. Stretching RPVs or treating with leptin for 24 h significantly lowered G-actin levels and increased F-actin compared to the controls (Figures 3B,C,E,F), consistent with the Western blot findings.

### Mechanical Stretch Induces ROS Production in VSMCs

**FIGURE 4:**
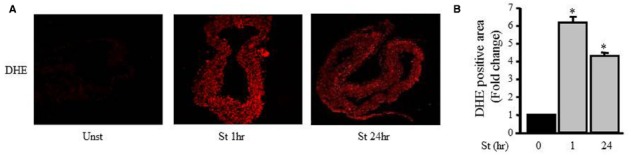
**Mechanical stretch-induced ROS production. (A)**. Representative confocal microscopic images of RPV wall stained with DHE. **(B)**. Fluorescence intensity measurements of DHE positive area of the mechanically stretched RPVs for 1 or 24 h (*n*=4). *p < 0.05 versus unstretched (0 h).

Figure 4A shows that the DHE fluorescence level (red fluorescent signal; ROS) in the unstretched RPV (control) was low while the DHE fluorescence intensity significantly increased after 1 and 24 h of mechanical stretch (six- and fourfolds, respectively) compared to unstretched RPV (Figures 4A,B).

**FIGURE 5:**
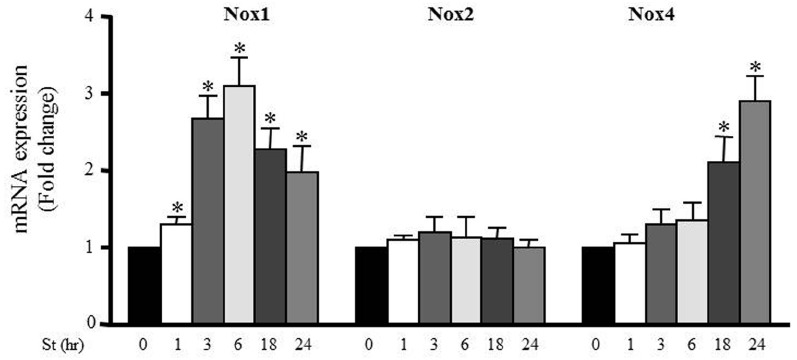
**Mechanical stretch-induced Nox expression.** Effect of mechanical stretch on the mRNA expression of Nox1, Nox2, and Nox4. (*n*=8–10). *p < 0.05 versus control (0 h).

Accumulating evidence suggests that NADPH oxidase (Nox) activity and expression have an important role in ROS formation in various cells types including VSMCs (Clempus and Griendling, 2006; Lyle and Griendling, 2006). To investigate the effect of mechanical stretch on Nox expression, RPVs were stretched for 1, 3, 6, 18, or 24 h and the mRNA expressions of Nox1, Nox2, and Nox4 were analyzed using qPCR. Nox1 and Nox4 mRNA expressions were increased after mechanical stretch, while Nox2 mRNA expression was not affected by mechanical stretch (Figure 5). These data indicate that Nox1 and Nox4 may be important in producing ROS after 24 h of stretch, but perhaps not so much after 1 h. On another hand, Nox2 expression had no role in mediating mechanical stretch-induced ROS formation.

### ROS Formation is Mediated by Leptin and the RhoA Pathway

**FIGURE 6:**
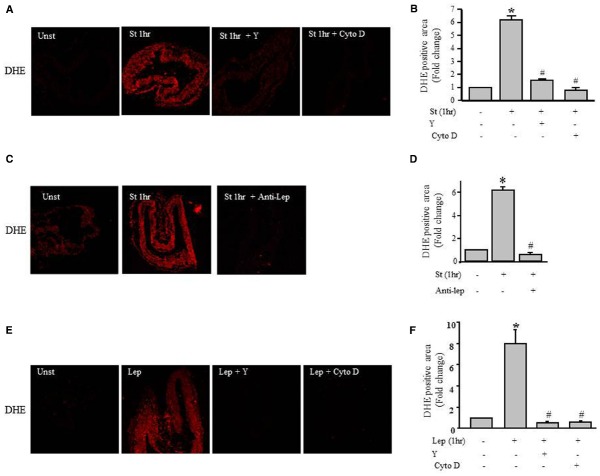
**Role of the RhoA/ROCK pathway in leptin- / mechanical stretch-induced ROS formation.** Effect of Y-27632 (Y; 10 μmol/L) or cytochalasin D (Cyto D; 1 μmol/L) on mechanical stretch- **(A)** and leptin- **(E)** induced ROS formation. **(C)**. Effect of inhibition of leptin by anti-leptin antibody (Anti-Lep) on mechanical stretch-induced ROS formation. Fluorescence intensity measurements of DHE positive area of the mechanically stretched **(B,D)** and leptin-induced **(F)** RPVs treated with Anti-Lep, Y, or Cyto D (*n*=3). *p < 0.05 versus unstretched (Unst). #*p* < 0.05 versus stretched 1 hr (St 1 h).

To gain insight into the mechanism underlying the mechanical stretch-induced increase in ROS production, stretched RPVs for 1 h were pretreated with the ROCK inhibitor Y-27632 compound (10 μmol/L) and the actin depolymerization agent cytochalasin D (1 μmol/L). DHE fluorescence revealed that treatment with either Y-27632 compound or cytochalasin D significantly decreased mechanical stretch-induced ROS formation (Figures 6A,B), suggesting the involvement of the RhoA/ROCK pathway and the importance of an intact cytoskeleton in the production of ROS induced by mechanical stretch.

To investigate whether leptin synthesis is involved in mechanical stretch-induced ROS formation, RPVs were pretreated anti-leptin antibody for 1 h followed by mechanical stretch for 1 h. Figures 6C,D show that anti-leptin antibody significantly prevented mechanical stretch-induced ROS production, indicating the significant role of leptin in mechanical stretch-induced ROS generation.

To gain more information about the effect of exogenous leptin on ROS formation, RPVs were treated with 3.1 nmol/L leptin for 1 h, equivalent to leptin concentrations in obesity. Figures 6E,F shows that leptin treatment significantly increased ROS formation, whereas pretreatment with Y-27632 or cytochalasin D significantly attenuated leptin-induced ROS production. These data show the involvement of Rho/ROCK pathway and intact actin cytoskeleton in leptin-induced ROS formation.

**FIGURE 7:**
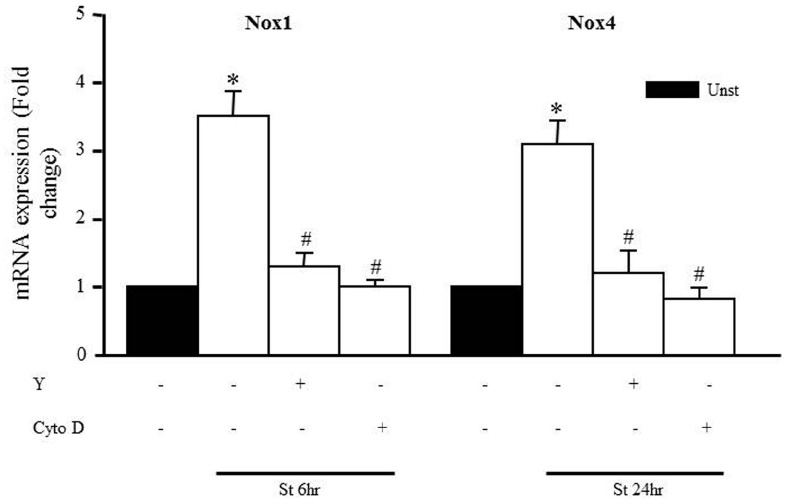
**Role of the RhoA/ROCK pathway and intact cytoskeleton on Nox expression.** Effect of Y-27632 (Y; 10 μmol/L) or cytochalasin D (Cyto D; 1 μmol/L) on mechanical stretch-induced Nox1 and Nox4 gene expression using qPCR analysis. (*n*=8–10). *p < 0.05 versus unstretched. #*p* < 0.05 versus stretched.

We also assessed whether Y-27632 and cytochalasin D could prevent the mechanical stretch-induced upregulation of Nox1 and Nox4 mRNA expression using qPCR analysis after 6 and 24 h, respectively (the peaks of mRNA upregulation). Figure 7 shows that upregulation of Nox1 and Nox4 induced by mechanical stretch was attenuated when RPVs were pre-treated with Y-27632 or cytochalasin D, indicating the involvement of ROCK activation and an intact actin cytoskeleton in mechanical stretch-induced Nox1 and Nox4 overexpressions.

### Leptin Induces GATA-4 Phosphorylation and Nuclear Translocation

**FIGURE 8:**
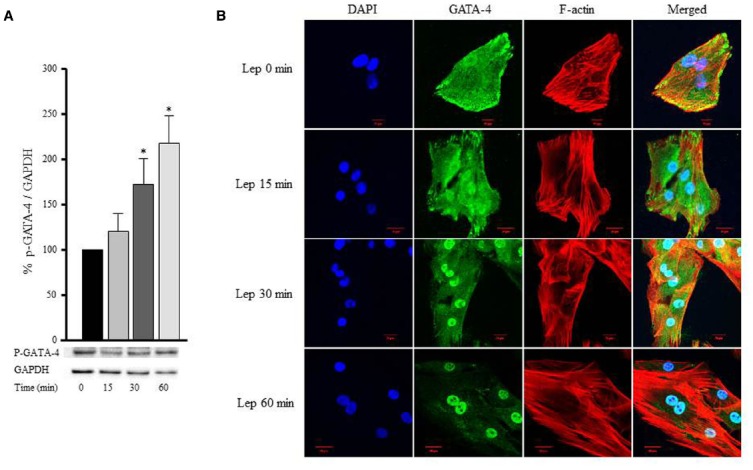
**Leptin-induced GATA-4 phosphorylation and nuclear translocation.** RASMC were treated with leptin (3.1 nmol/L) for 15, 30, and 60 min followed by Western blotting **(A)** or laser confocal microscopic analysis **(B)** for GATA-4 detection. Protein lysates were immunoblotted using anti-p-GATA-4 antibody to detect GATA-4 phosphorylation using Western blot **(A)**. For immunostaining to detect GATA-4 translocation to the nucleus, GATA-4 antibody was visualized by CruzFluor 488-conjugated secondary antibody (second panel; green). Acti-stain 555 phalloidin stained F-actin (third panel; red), while DAPI stained the nuclei blue (first panel; blue). The overlay of DAPI, GATA-4, and F-actin (merged) is shown in the right panel.

GATA-4 is a transcription factor that translocates from the cytoplasm to the nucleus upon activation, and in turn upregulates hypertrophic gene expression. To study whether the hypertrophic effect of leptin was mediated by GATA-4 phosphorylation and nuclear translocation, RASMC were treated with leptin (3.1 nmol/L) for 15, 30, and 60 min, followed by Western blotting or immunostaining to mark GATA-4 proteins. Figures 8A,B shows that GATA-4 phosphorylation and nuclear translocation were markedly increased by 30 and 60 min of leptin treatment. Thus, leptin mediates vascular remodeling, at least in part, by a GATA-4-dependent mechanism.

### GATA-4 Nuclear Translocation is Dependent on the RhoA/ROCK Pathway and an Intact Cytoskeleton

**FIGURE 9:**
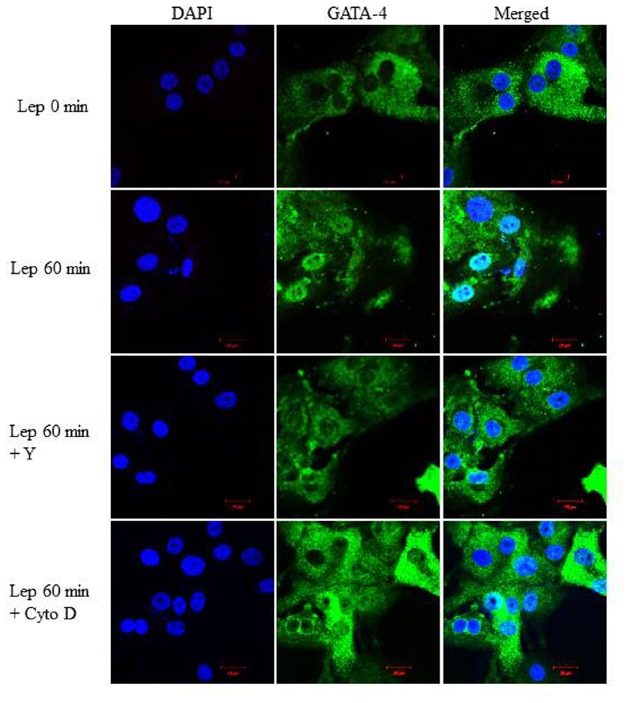
**Role of the RhoA/ROCK pathway in leptin-induced GATA-4 translocation.** Representative confocal microscopic images of RASMC to study the effect of Y-27632 (Y; 10 μmol/L) or cytochalasin D (Cyto D; 1 μmol/L) on leptin-induced GATA-4 translocation. DAPI stained the nuclei (left panel; blue) and GATA-4 was detected using CruzFluor 488-conjugated secondary antibody (middle panel; green). The overlay (merged) is shown in the right panel. GATA-4 translocation in response to leptin was abolished by Y and Cyto D.

To examine whether the RhoA/ROCK pathway is involved in mediating GATA-4 nuclear translocation in response to leptin, RASMC were pre-treated with Y-27632 (10 μmol/L) or cytochalasin D (1 μmol/L) followed by leptin treatment for 60 min. GATA-4 nuclear translocation was noticeably abolished by treatment with either Y-27632 or cytochalasin D (Figure 9), indicating that the RhoA/ROCK pathway and an intact cytoskeleton are crucial for leptin-induced GATA-4 translocation to the nucleus.

### ROS Depletion Inhibits GATA-4 Nuclear Translocation

**FIGURE 10:**
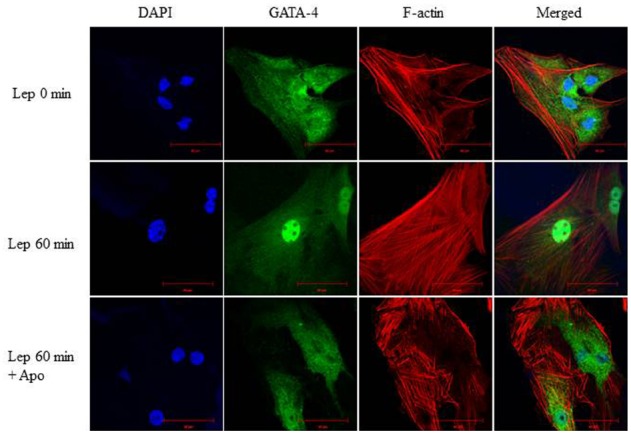
**Involvement of ROS in leptin-mediated GATA-4 nuclear translocation.** Effect of the NADPH oxidase inhibitor apocynin (1 μmol/L) on leptin-induced GATA-4 nuclear translocation in RASMC. GATA-4 antibody was visualized by CruzFluor 488-conjugated secondary antibody (second panel; green) while Acti-stain 555 phalloidin was used to stain F-actin (third panel; red). DAPI stained the nuclei blue (first panel; blue). The overlay of DAPI, GATA-4, and F-actin (merged) is shown in the right panel. ROS depletion inhibited GATA-4 nuclear translocation.

To study whether there is an interaction between ROS and GATA-4, the Nox inhibitor apocynin (1 μmol/L) was added to RASMC to deplete ROS, followed by leptin treatment. RASMC treated with apocynin and leptin for 60 min exhibited a marked reduction in GATA-4 nuclear translocation compared to leptin-treated cells alone (Figure 10), indicating that ROS are upstream to GATA-4 translocation.

## Discussion

Understanding the molecular mechanisms involved in cardiovascular disease during obesity and hypertension is crucial in order to identify novel therapeutic targets for treating their associated-vascular and cardiac disorders. Obesity is associated with high levels of the circulating hormone leptin (hyperleptinemia), which in turn is responsible for several cardiovascular diseases. The exact mechanism(s) by which leptin is associated with hypertension and the progression of cardiovascular diseases remains to be investigated as discussed in this work.

**FIGURE 11:**
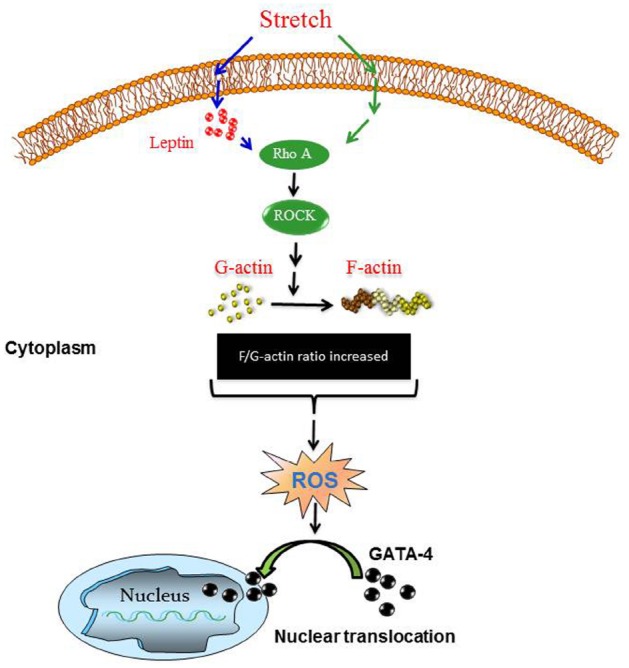
**Proposed scheme for mechanical stretch-induced VSMC hypertrophy through leptin synthesis, RhoA/ROCK pathway, ROS formation, and GATA-4 nuclear translocation.** Please see Discussion for description.

The major findings in the present study include: (1) Identification of a direct effect of the mechanical stretch model (mimicking hypertension) on endogenous leptin synthesis in VSMCs. (2) Involvement of Nox1 and Nox4 but not Nox2 expression in mechanical stretch-induced ROS formation in VSMCs. (3) Leptin induces ROS formation in VSMCs, in a RhoA/ROCK-dependent manner. (4) Leptin induces GATA-4 phosphorylation and nuclear translocation through the RhoA/ROCK pathway and ROS, as summarized in Figure 11.

Rat portal vein organ culture was used in this study to evaluate the effect of mechanical stretch on vascular remodeling. This *ex-vivo* model was used since it has been well-characterized in a number of recent publications related to the hypertrophic effect of mechanical stretch (Zeidan et al., 2000, 2003a,b, 2004, 2005; Ren et al., 2010; Turczynska et al., 2012, 2013).

Because of its myogenic vasomotion, the RPV has been used in the development of vasoactive drugs as an analog of small pre-capillary resistance vessels (Ljung, 1990). Moreover, the longitudinally oriented musculature in the RPV makes it an ideal blood vessel for investigating the effect of mechanical stress on muscle hypertrophy by stretching it by weight loading rather than perfusion. In addition, mechanical stretch maintained the differentiated status of the VSMCs in this *ex-vivo* model as evidenced by a marked increase in SM22 synthesis (differentiation marker for VSMCs) and muscle contractility, unlike VSMCs in cell culture (Zeidan et al., 2003b). Moreover, the effect of increased RPV pressure (*in vivo*) due to partial occlusion in the portal vein has been implicated in VSMC hypertrophy (Malmqvist and Arner, 1988, 1990). Many signaling complexes act as sensors and transducers of mechanical stretch such as growth factors, ROS, nuclear factor kappaB, and Na^+^/H^+^ exchanger isoform 1 (Dzau, 1993; Koller, 2002; Lemarie et al., 2006; Paravicini and Touyz, 2006; Haga et al., 2007). We have previously shown that mechanical stretch stimulated VSMC hypertrophy via the activation of MAPK pathways, RhoA/ROCK pathways, and actin cytoskeleton remodeling (Zeidan et al., 2000, 2003a,b, 2004, 2005).

Many studies have shown a clear relationship between plasma leptin levels and hypertension in different animal models (Hiraoka et al., 1997; reviewed, by Stenvinkel, 2000) and in humans (Agata et al., 1997; Uckaya et al., 1999). However, the mechanism by which hypertension increases plasma leptin levels is still not clear. Plasma levels of leptin, in general, are higher in essential hypertensive patients (not obese) compared to controls (Agata et al., 1997; Uckaya et al., 1999). Indeed, we have previously found that mechanical stretch enhances the release of leptin from VSMCs to culture media and increases leptin mRNA expression after 1, 2, and 3 days of stretching along with vascular remodeling (Zeidan et al., 2005).

The current study sought to delineate the possible role of increased mechanical stretch (hypertension) on leptin protein synthesis in VSMCs and on early time points of leptin release (1, 3, 6, 18 h). Interestingly, we have found that significant alterations in leptin protein expression occurred after only 1 h of mechanical stretch coupled with lower intracellular leptin levels 24 h post mechanical stretch. The hypothesis for this decrease in the intracellular leptin levels after 24 h is that leptin was secreted outside the VSMCs and that the biosynthesis of leptin itself was downregulated; the underlying mechanism needs to be further investigated. However, these data highlighted the critical role of leptin in mechanical stretch induced-VSMC hypertrophy as discussed in previous findings from our lab (Zeidan et al., 2003a).

To further validate the above findings, CM taken from stretched RPVs was shown to induce RPV hypertrophy while CM from unstretched RPVs had no effect on RPV hypertrophy, indicating the presence of hypertrophic related-protein(s) in CM as shown in Figure 2. Adding the anti-leptin antibody to CM taken from stretched RPVs inhibited the hypertrophic effect of CM, indicating the important role of secreted leptin in mediating CM-induced RPV hypertrophy.

As for the dynamics of actin cytoskeleton, our work shows that mechanical stretch increases actin polymerization as reflected by an increase in the F-actin to G-actin ratio by 5.6 fold, while leptin increases it by 1.5 fold (Figure 4). The reported results are in line with the idea that leptin decreases the levels of G-actin with respect to F-actin via the RhoA/ROCK/p-cofilin pathway.

Among the aims of this study was to investigate the role of ROS formation in mechanical stretch-induced VSMC remodeling. ROS has several implications on cardiovascular functions (Zafari et al., 1998; Touyz and Schiffrin, 1999), including vascular remodeling. In this study, ROS formation was upregulated 1 h post mechanical stretch. Of interest, treating RPVs with exogenous leptin exhibited similar results to mechanical stretch, while inhibiting leptin synthesis in turn inhibited mechanical stretch-induced ROS formation. These data indicate that ROS formation is induced through leptin synthesis as shown in Figure 6. Taken together, our data strongly suggest the important pathophysiological roles of hypertension and hyperleptinemia for increased ROS production in VSMCs.

Moreover, this study shows a link between ROS production and the RhoA/ROCK pathway activation. ROCK inhibition and depolymerization of the actin cytoskeleton significantly lowered both leptin-/mechanical stretch-induced ROS formation, thereby placing ROS formation downstream to RhoA/ROCK (Figure 6).

Moreover, we analyzed the effect of mechanical stretch on the expression pattern of different NADPH oxidases (Nox1, Nox2, Nox4). Mechanical stretch upregulated Nox1 and Nox4 expressions but not Nox2, indicating the important role of Nox1 and Nox4 in mechanical stretch/hypertension-induced vascular remodeling. Nox1 expression increased significantly after 1 h of mechanical stretch and remained high throughout until 24 h, indicating that Nox1 is perhaps an early response NADPH oxidase to stretch (Szöcs et al., 2002). Nox4 expression increased significantly after 18 h of stretch, suggesting that it is responsible for the late (24 h) production of ROS in response to stretch. Given these time points, we concluded that Nox1 and Nox4 are responsible for generating ROS in response to 24 h of stretch, but not at 1 h of stretch. Nox2 expression did not change in response to stretch and thus not responsible for the increase in ROS formation after stretch. It is interesting to note that a recent study by Byrne et al. reported that Nox2 expression and activation is not critical for pressure overload-induced cardiac hypertrophy (Byrne et al., 2003; Maytin et al., 2004). Moreover, the importance of Nox4 has been shown in pressure overload-induced left ventricular hypertrophy (Byrne et al., 2003). Our results have shown that Nox1 and Nox4 upregulation was attenuated by both ROCK inhibitor and actin depolarization agent, further suggesting an important role for the RhoA/ROCK pathway in downstream ROS formation.

Increased wall tension results in hypertrophy, which begins as a compensatory response, but is actually detrimental. The role of GATA-4 in promoting in hypertrophy has been well established in the cardiac system, since it is a transcription factor that activates hypertrophic gene expression like β-MHC, c-fos, c-jun, c-myc, BNP, and ANF (Chien et al., 1991; Liang and Gardner, 1999; Saadane et al., 1999; Babu et al., 2000). We already know that leptin induces VSMC hypertrophy (Zeidan et al., 2005), and this current study reveals a mechanism of leptin-induced VSMC hypertrophy (Figure 3). Furthermore, our present data indicate that leptin promotes the phosphorylation and nuclear translocation of GATA-4, and that this leptin-induced GATA-4 activation is inhibited by the ROCK inhibitor and actin depolymerization agent (Y-27632 and cytochalasin D respectively). Since leptin and the RhoA/ROCK pathway are implicated in VSMC hypertrophy (Yamakawa et al., 2000; Zeidan et al., 2005), we believe that GATA-4 activation and translocation plays a hypertrophic role in the vascular system as shown by these new findings. Moreover, the role of GATA-4 has been linked in the vascular system by regulating cardiac angiogenesis (Heineke et al., 2007) and embryonic angiogenesis in the vascular plexus (Torregroza et al., 2012). The contribution of ROS to GATA-4 activation in response to leptin has been studied. The NAPDH oxidase inhibitor apocynin was used to inhibit ROS formation (Pi et al., 2013; Schroeter et al., 2013), followed by analysis of GATA-4 nuclear translocation. ROS depletion markedly abolished GATA-4 nuclear translocation in response to leptin. Therefore, these data indicate that ROS may activate GATA-4 and induce GATA-4 nuclear translocation.

In conclusion, this study highlighted the molecular mechanisms involved in mechanical stretch-induced VSMC hypertrophy and the critical involvement of endogenous leptin, ROS formation, and GATA-4 nuclear translocation. Based on our findings in this study, we were able to construct a putative pathway of the mechanism mediating the above changes of mechanical stretch/leptin cross-talk as depicted in Figure 11. As shown, mechanical stretch acts as an upstream regulator of ROS formation and GATA-4 nuclear translocation through leptin synthesis, RhoA/ROCK activation, and F/G-actin ratio changes (please refer to Figures 1, 3, 4, and 6)

Further research should be done in order to provide a more detailed picture of the mechanisms of action. The links between the different signal transducers may provide a helpful approach in developing potential therapeutic strategies to attenuate the harmful effects of hypertension and leptin on vascular remodeling.

### Conflict of Interest Statement

The authors declare that the research was conducted in the absence of any commercial or financial relationships that could be construed as a potential conflict of interest.
